# Impact of age and vaccination history on long-term serological responses after symptomatic *B. pertussis* infection, a high dimensional data analysis

**DOI:** 10.1038/srep40328

**Published:** 2017-01-16

**Authors:** Inonge van Twillert, Axel A. Bonačić Marinović, Betsy Kuipers, Jacqueline A. M. van Gaans-van den Brink, Elisabeth A. M. Sanders, Cécile A. C. M. van Els

**Affiliations:** 1 Centre for Infectious Disease Control, National Institute for Public Health and the Environment (RIVM), Bilthoven, the Netherlands; 2 Department of Immunology and Infectious Diseases, Wilhelmina Childrens Hospital, University Medical Center Utrecht, Utrecht, the Netherlands

## Abstract

Capturing the complexity and waning patterns of co-occurring immunoglobulin (Ig) responses after clinical *B. pertussis* infection may help understand how the human host gradually loses protection against whooping cough. We applied bi-exponential modelling to characterise and compare *B. pertussis* specific serological dynamics in a comprehensive database of IgG, IgG subclass and IgA responses to Ptx, FHA, Prn, Fim2/3 and OMV antigens of (ex-) symptomatic pertussis cases across all age groups. The decay model revealed that antigen type and age group were major factors determining differences in levels and kinetics of Ig (sub) classes. IgG-Ptx waned fastest in all age groups, while IgA to Ptx, FHA, Prn and Fim2/3 decreased fast in the younger but remained high in older (ex-) cases, indicating an age-effect. While IgG1 was the main IgG subclass in response to most antigens, IgG2 and IgG3 dominated the anti-OMV response. Moreover, vaccination history plays an important role in post-infection Ig responses, demonstrated by low responsiveness to Fim2/3 in unvaccinated elderly and by elevated IgG4 responses to multiple antigens only in children primed with acellular pertussis vaccine (aP). This work highlights the complexity of the immune response to this re-emerging pathogen and factors determining its Ig quantity and quality.

*Bordetella pertussis* causes the respiratory infectious disease ‘whooping cough’ (pertussis) that has remained endemic even in populations with high vaccination coverage, affecting all age groups^1^,^2^. Protective immunological memory to *B. pertussis* is not long-lasting, as observed after natural infection and immunization with whole cell pertussis vaccines (wP)^3^, but is apparently even shorter-lived after use of current acellular pertussis vaccines (aP)^4^,^5^. So far, no unambiguous correlates of protection for pertussis have been established, though both humoral and cellular adaptive immunity are likely engaged^6^,^7^,^8^. Infection involves attachment of *B. pertussis* to cells in the upper and lower respiratory tract, mediated by adhesins like filamentous hemagglutinin (FHA), pertactin (Prn) and fimbriae (Fim2 and/or Fim3). Consequently, antibodies with adhesin specificity and opsonizing and bactericidal effector function, may provide protection against colonization^9^,^10^. Pertussis toxin (Ptx), an important virulence factor and unique for *B. pertussis*, is implied in the pathogenesis of clinical pertussis symptoms^11^,^12^ and capable of suppressing and modulating host immune responses^13^,^14^,^15^. Low levels of pertussis toxin (Ptx) specific immunoglobulins (Ig) have been shown to correlate with susceptibility to disease^16^,^17^. At mucosal surfaces, secretory (dimeric) IgA is the first line of defence against *B. pertussis*. In serum, (monomeric) IgA is the second most prevalent antibody type after IgG. Serum IgA, though a poor activator of complement, can trigger effector functions that may extinguish bacteria^18^. IgG, the most abundant antibody type in peripheral blood consists of four IgG subclasses: IgG1is the dominant subtype, followed by IgG2, IgG3 and IgG4^19^,^20^. These subclasses have distinct effector functions, mediated by differences in the Fc part of the Ig molecule. Human IgG3 is a potent pro-inflammatory antibody, opsonising invading microorganisms and the best activator of the complement system, followed by IgG1^21^. In adults, IgG2 is associated with the (T cell independent) response to bacterial capsular polysaccharides^22^. Infants, having still weak T cell independent responses, are known to have less production of IgG2^23^ though IgG2 production can be stimulated by polysaccharide vaccines^24^. IgG4, like IgE, can be induced by allergens. Levels of IgG4 are also often measured after repeated exposure to antigen in a non-infectious setting^21^,^25^.

The dynamics of the serological response to *B. pertussis*, as it has been studied after infection or vaccination, is generally described by group level statistics of antibody levels at distinct time intervals after an immunizing event^26^,^27^,^28^. This approach however is limited when time of sampling is not harmonized, follow up is restricted and if large inter-subject variation in responsiveness is involved, the latter being the case for *B. pertussis* humoral responses.

Mathematical models can accommodate for these events and kinetic modelling assuming first order (simple exponential) decay of IgG responses against mostly only Ptx, has been described^29^,^30^,^31^,^32^. Human seroresponses to *B. pertussis* bacteria, however involve multiple specificities and Ig types. Also, decay curves of Ig levels may rather be biphasic, reflecting the contribution of both rapidly declining short-lived plasma cells and slowly declining long-lived plasma cells to the Ig production^33^. Such biphasic decay in pertussis serology was indeed earlier suggested by two Scandinavian studies, following IgG responses to Ptx both in vaccinees and pertussis cases^27^,^28^. The aim of our study was to substantially enrich our knowledge on the waning of multiple parallel Ig responses to *B. pertussis* across age groups by modelling of data assuming biexponential decay. Using samples from a large cohort of clinical pertussis cases and ex-cases of all ages from the SKI-study^34^,^35^, and multiplex technology, we created a comprehensive database of levels of various Ig (sub) classes to multiple *B. pertussis* specific antigens. Subsequently, a biexponential decay model was applied to extract and compare essential features of the ongoing host response, such as immediate levels, decline patterns, antigenic breadth and effector types of Ig, in a systematic way. We hypothesize that features will vary between cohorts of different ages, having diverse vaccination and/or exposure histories. Better understanding of how resistance is built up to this re-emerging pathogen in the overall population is essential to support improved vaccine strategies in the future.

## Methods

### Ethics

Participants were symptomatic (ex-) pertussis cases included in a Dutch cross-sectional observational study conducted between January 2008 and December 2012 (Specifieke Kinkhoest Immuniteit; SKI)^34^,^35^. This study was approved by the accredited Review Board STEG followed by management of the METC UMC Utrecht (CCMO nr: NL16334.040.07). All participants provided written informed consent. Written informed consent for minor participants was provided by both parents or guardians of participants. This study was conducted in compliance with the principles of the Declaration of Helsinki.

### Study populations

**Figure 1:**
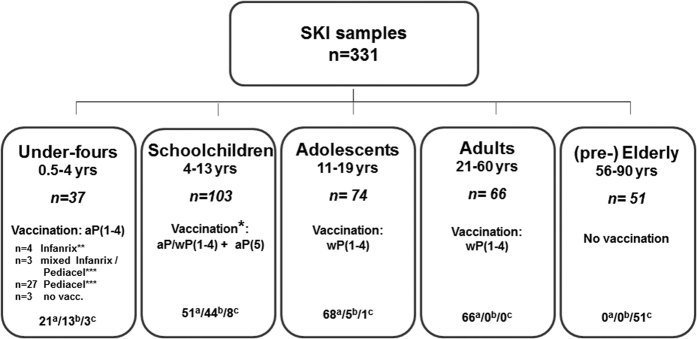
Flowchart of SKI samples stratified according to age and vaccination status. aP(1–4): acellular pertussis vaccination doses 1–4 [either Infanrix (GSK; GlaxoSmithKline) threevalent pertussis vaccine or Pediacel (SP, SanofiPasteur) five-valent pertussis vaccine], aP(5): acellular pertussis preschool booster dose 5; wP(1–4): whole cell pertussis vaccination doses 1–4 (Netherlands Vaccine Institute or predecessor). According to the Dutch National Immunization Program (NIP) pertussis vaccine primary doses 1–4 are given at 2, 3, 4, and 11 months of age, and the preschool booster dose 5 is given at 4 years of age, for all birthcohorts since 2001. a =  number of samples where pertussis infection had taken place after pertussis vaccination, b =  number of samples where the pertussis infection had preceded pertussis vaccination, c =  number of samples of (voluntarily) unvaccinated (ex) pertussis cases, (pre-) Elderly were born before the implementation of the National Immunization Programme. *Vaccination details of the schoolchildren are given in Supplementary Table 1. **Infanrix-IPV-Hib (GSK): containing threevalent pertussis vaccine (Ptx: 25 ug, FHA: 25 ug, Prn: 8 ug). ***Pediacel (Sanofi Pasteur): containing five-valent pertussis vaccine (Ptx: 20 ug, FHA: 20 ug, Prn: 3 ug, Fim2/Fim3: 5 ug).

289 (Ex-) pertussis cases of all ages, who had presented with clinical symptoms, were physician-diagnosed with pertussis (based on standardized PCR-, culture- or serological diagnostic assays in accredited laboratories). For the study blood was drawn at a known time interval, ranging from 0.2–191.5 months, after date of diagnosis. Participants (male; n = 145 and female; n = 186) were classified into five age groups (Fig. 1), depending on both age and history of childhood pertussis vaccination (Fig. 1): under-fours, schoolchildren, adolescents, adults, and (pre-) elderly. Forty-two cases donated two blood samples, the first 0.4–2.1 (median 0.8) months after diagnosis, the second 13.5–56.9 (median 40.1) months after diagnosis, totalling the number of samples to 331. As indicated in Fig. 1, for 269 blood samples, the date of diagnosis was the last known date of contact with pertussis antigens; for the 62 other samples, a pertussis vaccination had taken place between their clinical pertussis episode and blood withdrawal, and for these samples date of last vaccination was computed as last known date of contact with pertussis antigens. For unmodelled data analysis, a smaller group of subjects was stratified based on the time after last exposure to pertussis antigens and date of blood sampling: [≤9 months] and [9–48 months], based on earlier work^34^ and as indicated.

### Blood sampling

Venous blood samples were collected in vacutainer cell preparation (CPT) tubes. Plasma was isolated using standard procedures and stored at −80° until testing.

### Antigens

Pertussis Toxin (Ptx) and Filamentous Hemagglutinin (FHA) were obtained from Kaketsuken (Japan). P.69 pertactin (Prn) and Fimbriae types 2 and 3 mix (Fim2/3) were obtained from Sanofi, formerly Aventis Pasteur (France). *B. pertussis* specific Outer Membrane Vesicles (OMV) were generated in-house, as described elsewhere^36^.

### Multiplex immuno assay

Levels of Ig (sub) classes against pertussis antigens Ptx, FHA, Prn, FIM2/3 and OMV were measured in plasma samples applying an optimized in-house multiplex immunoassay (MIA) described earlier^34^,^37^ with some modifications. For each antigen fluorescently labelled microspheres were used with a distinct bead region (Bio-Rad Laboratories, Hercules, USA). Activated beads were coupled to antigen, mixed to form a heptaplex bead platform, and then incubated with serial dilutions of reference sera or diluted plasma samples. Plasma samples were prepared in the dilutions 1/300 and 1/3600 for IgG and in 1/50 and 1/500 dilutions for measurement of IgG subclasses and IgA, respectively, in parallel series. As a reporter antibody Goat anti-human IgG, R-PE (Jackson ImmunoResearch, USA) and Goat anti-human-IgA-PE (Southern Biotech, USA) were used for detecting IgG and IgA levels, respectively. IgG subclass measurement required two detection steps; Mouse anti-human IgG1, IgG2, IgG3 or IgG4 (all from Invitrogen, USA), followed by Goat anti-Mouse IgG-PE (Jackson ImmunoResearch, USA). The International WHO pertussis standard (NIBSC 06/140) was used as reference for IgG and IgA levels against Ptx, FHA and Prn (International units, IU/ml) and for IgA against Fim2/3 and OMV (arbitrary units, AU/ml). Human Normal Immunoglobulin solution for infusion (KIOVIG, Baxalta, (formely Baxter AG), Vienna, Austria) was used as an in-house reference to measure levels of IgG against Fim2/3, and of IgG subclasses against all antigens, in arbitrary units per ml (AU/ml) based on observed relative magnitudes of Fluorescence Intensity (FI). Analysis was performed with a Bio-Plex 200 in combination with Bio-Plex Manager software version 5.5 (Bio-Rad Laboratories, USA). Some measurements corresponded to values below the lower limit of detection (LLD) (lowest measurable concentration within the linear range of the reference curve) and these were censored. For graphical purposes these measurements were assigned the LLD divided by two. For modelling procedures these measurements were considered censored below the LLD and assumed to follow the distribution patterns from the known data in the dataset. Those few measurements corresponding to values above the higher limit of detection (HLD) (when the highest sample dilution fell above the linear range of the reference curve), were assigned values based on bridging to samples having corresponding FIs above the HLD for their least diluted sample dilution, but having FIs in the linear range of the reference curve for their most diluted sample dilution. The relative contribution of an IgG subclass response against an antigen to the total IgG response was calculated as the following percentage: [FI of the specific IgG subclass response]/[summed FIs of all four specific IgG subclass responses] * 100%.

### Biexponentional decay model

**Figure 2:**
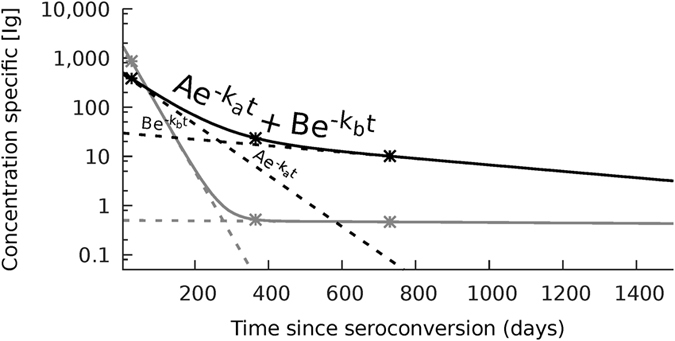
Model of specific Ig concentration as a function of time since last antigen contact. The biexponential decay model describes the waning episode of an Ig response with the shape Ae^−k(a)t^ + Be^−k(b)t^. One component represents an acute fast-decaying Ig response, with initial concentration A and decay rate k_a_; and the other component represents a slow-decaying maintenance Ig response, with initial concentration B and decay rate k_b_. At each given time point the model-predicted value of the response is the sum of both components. The figure shows two examples of the final curve, one grey and one black line, which incorporate the two underlying A and B components.

The waning episode of the Ig responses was assumed to be biphasic and described by a biexponential decay model with the shape **Ae**^**−k**^_**a**_^**t**^** + Be**^**−k**^_**b**_^**t **^33. One component represents an acute fast-decaying Ig response, with initial concentration A and decay rate k_a_; and the other component represents a slow-decaying maintenance Ig response, with initial concentration B and decay rate k_b_. At each given time point the model-predicted value of the response is the sum of both components (Fig. 2). For each measured Ig response distributions for parameters A, B, k_a_ and k_b_ were extracted from the Ig dataset by means of a Bayesian approach with Monte Carlo simulations, for age groups combined and for age groups separately. Using the determined A, B, k_a_ and k_b_ for each response, quantiles (97.5^th^, 50^th^ and 2.5^th^ percentiles) of the Ig concentrations were predicted as a function of time since last pertussis antigen contact. Estimates of the 50^th^ percentiles (medians) of each Ig response were used for comparative analysis between age groups or time points. Serodynamic trends of Ig responses were analysed using data extracted at 6 arbitrary points in time, i.e. 28, 70, 140, 365, 730 and 1095 days after last pertussis antigen contact. The model does not capture the initial rise of the Ig response and day 28 was taken to predict Ig peak levels. Of the 289 SKI cases, 42 cases donated a second blood sample; modelling exclusive of these longitudinal samples did not indicate a confounding effect. Three waning factors calculated by a concentration ratio were used to express the decay of Ig responses: (a) week-10 waning factor: [Ig]t = 28/[Ig]t = 70, (b) year-2 waning factor: [Ig]t = 28/[Ig]t = 730, (c) year-3 waning factor: [Ig]t = 28/[Ig]t = 1095, with t in days and [Ig] being the 50^th^ Ig percentiles.

**Figure 3:**
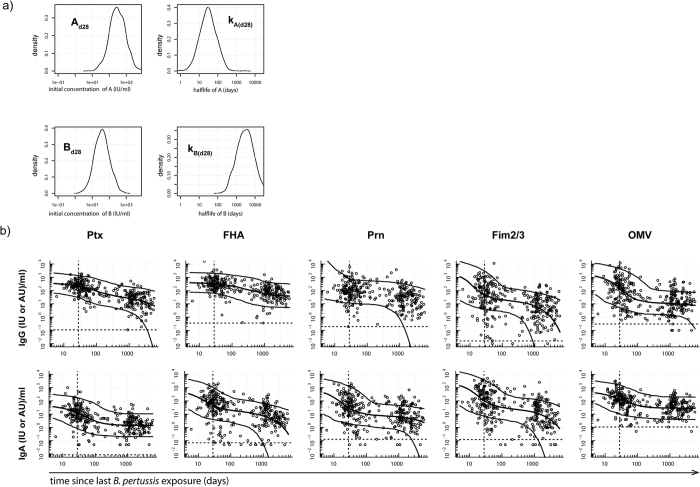
Measured Ig levels and model parameters in IgG and IgA datasets of all SKI cases. An example is given of density plots showing distributions for the model parameters (A, B, k_a_ and k_b_) describing the measured IgG-Ptx dataset of all SKI cases: A and B components (at day 28, in IU/ml) (left panels), and their respective half-lives (days) (right panels) (**a**). Dot plots show measured IgG and IgA levels to Ptx, FHA, and Prn (in IU/ml) and to Fim2/3 and OMV (in AU/ml) of all SKI cases as a function of time after exposure (in days, logarithmic scale). Open circles represent measured IgG and IgA levels, lines indicate 5^th^, 50^th^ (medians) and 95^th^ percentiles of the modelled responses. The IgG-OMV dot plot represents data of the IgG subclass IgG3 in AU/ml. (**b**).

**Figure 4:**
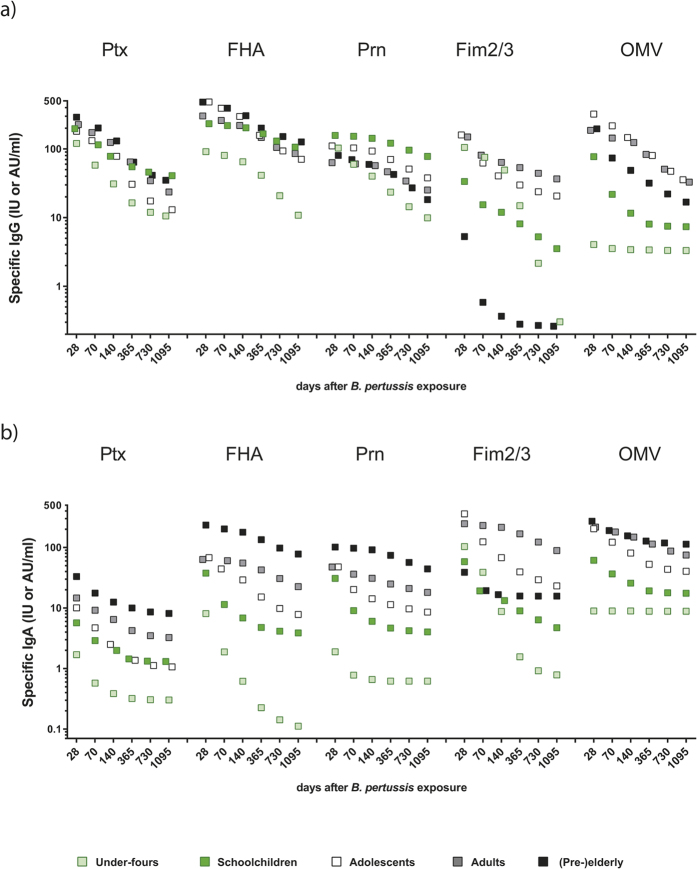
Predicted Ig levels against *B. pertussis* specific antigens stratified by age group. Median IgG (**a**) and IgA (**b**) values to *B. pertussis* specific antigens predicted by our biexponential decay model for six time points, i.e. day 28, day 70 (week 10), day 140 (week 20), day 365 (year 1), day 730 (year 2), day 1095 (year 3) (shown in days, non-linear time axis), for each age group, as indicated.

**Figure 5:**
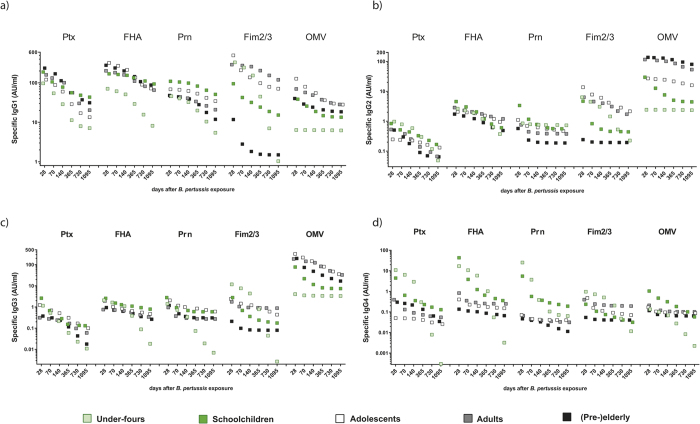
Impact of age group factors on IgG subclass levels against *B. pertussis* specific antigens. Median levels of IgG subclasses IgG1 (**a**), IgG2 (**b**), IgG3 (**c**) and IgG4 (**d**) to *B. pertussis* specific antigens predicted by our biexponential decay model for six time points, i.e. day 28, day 70 (week 10), day 140 (week 20), day 365 (year 1), day 730 (year 2), day 1095 (year 3) (shown in days, non-linear time axis), for each age group, as indicated.

**Figure 6:**
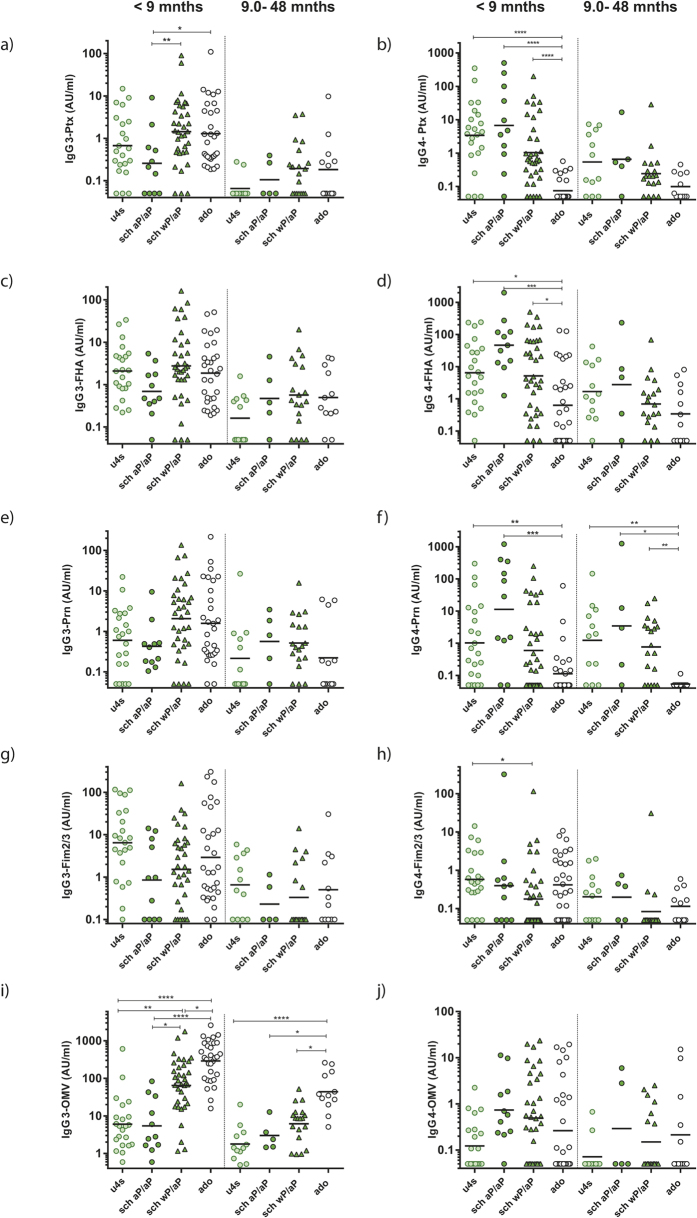
Priming with aP or wP vaccination leads to opposing trends in *B. pertussis* specific IgG3 and IgG4 responses after pertussis infection. Scatter dot plots of specific IgG3 (left panels) and IgG4 (right panels) concentrations of Ptx (**a**,**b**), FHA (**c**,**d**), Prn (**e**,**f**), Fim2/3 (**g**,**h**) and OMV (**i**,**j**) in under-fours (aP primed; u4s), schoolchildren (aP primed and aP boosted; sch aP/aP), schoolchildren (wP primed and aP boosted; sch wP/aP) and adolescents (wP primed, not boosted; ado) early (≤9 months) and late (9–48 months) after last known *B. pertussis* antigen exposure. Non vaccinated subjects were excluded. *p < 0.05; **p < 0.01; ***p < 0.001, ****p < 0.0001.

For modelling, data analysis and visualization of data software program “R”, version 3.1.2.and GraphPad Prism (GraphPad Software version 6.05) were used (Figs 1, 2, 3, 4 and 5). Statistical significance of differences in Ig levels between subcohorts (Fig. 6) were analysed with the nonparametric Kruskal-Wallis Test and Dunn’s Multiple comparison test. P values < 0.05 were considered statistically significant.

## Results

### High-dimensional dataset of serological responses in the waning episode after a *B. pertussis* infection

We acquired Ig data from 331 plasma samples from 289 (ex) pertussis cases of varying age and vaccination histories in different phases after a symptomatic pertussis infection using an in-house pentaplex MIA. In all samples we measured Ig levels against five pertussis antigens simultaneously, i.e. to Ptx, FHA, Prn, Fim2/3 and OMV. In parallel series six types of Ig levels to all antigens were measured, i.e. IgG, IgA, IgG1, IgG2, IgG3 and IgG4 levels (with the exception of the IgG-OMV series), creating a comprehensive dataset of 9599 Ig values. No interference of various antigen coupled bead regions was found between monoplex and pentaplex MIA.

### Applying a biexponential decay model that predicts *B. pertussis* specific Ig levels

To contend with the large individual variation in Ig responses in (ex-) pertussis cases, we used the data to perform Markov-chain Monte Carlo simulations in a biexponential longitudinal biomarker model. For each immune response the four model parameters, A, B, k_a_ and k_b_, were statistically predicted and employed in the model to capture the early phase of fast Ig decay followed by a second phase of slower Ig decay. As an example, distributions of A and B parameters at a given point in time (in IU/ml) and their respective half-lives, t_½_(A) and t_½_(B), are shown for the IgG-Ptx dataset (Fig. 3a). Analysis of residuals between the model predictions and the measured dataset indicate that the model follows the data accurately. Figure 3b depicts the measured levels of IgG and IgA of all clinical pertussis cases for all antigens, together with the 2.5^th^, 50^th^ and 97.5^th^ percentiles of the responses predicted by the model at any desired point in time. This allows to objectively compare immediate response ranges and kinetics of specific Ig levels after symptomatic pertussis between various antigens, Ig (sub) classes and age groups. IgG and IgA peak levels at day 28 were highly variable for all *B. pertussis* antigens; overall highest 2.5^th^ to 97.5^th^ percentile ranges at the peak of (total) IgG and IgA responses were found for IgG-Fim2/3 (4.1–3052.2 AU/ml) and IgA-Fim2/3 (7.0–3932.3 AU/ml), respectively, and lowest for IgG-Prn (11.6–755.5 IU/ml) and IgA-Ptx (0.8–176.2 IU/ml), respectively (as illustrated in Fig. 3).

### The model reveals differences in kinetics of *B. pertussis* antigen specific Ig responses: faster waning of IgA compared to IgG

To compare the overall pace of Ig waning between antigen specificities and Ig subtypes three waning factors were determined, for week 10, year 2 and year 3, respectively, based on model derived predicted median [Ig] values for the whole SKI-cohort (Table 1). Three years after a symptomatic pertussis infection, all *B. pertussis* specific IgG responses have declined, however not all antigen specificities wane to the same extent. IgG-Prn levels showed slowest waning, hardly measurable at week 10 (decay factor 1.2), and being decayed 2.0-fold and 2.4-fold at year 2 and 3, respectively, while IgG-Ptx levels had waned 1.7-fold at week 10, and had highest decay factors at year 2 and year 3, 7.1 and 8.3, respectively. Also, IgG-FHA and IgG-Fim2/3 Ig responses waned faster than Prn counterparts, having week 10 decay factors of 1.3 *versus* 2.0, year 2 decay factors of 4.6 *versus* 5.2, and year 3 decay factors of 5.6 and 7.7, respectively (Table 1).

In general, *B. pertussis* specific IgA plasma levels showed waning of greater magnitude than IgG counterparts, decay factors for all specific antigens after 10 weeks and after 3 years being 1.3–2 fold and 1.1–2 fold higher, respectively.

### The impact of age group factors on peak and maintenance levels of *B. pertussis* specific IgG and IgA

To investigate whether group factors such as age and vaccination history play a role in waning patterns of Ig responses after a clinical symptomatic infection we applied the biexponential decay model on the five age groups of the SKI cohort separately. Median IgG and IgA values for the *B. pertussis* specific antigens were thus predicted for six time points per age group (Fig. 4). Adults and (pre-) elderly reached higher peak levels for IgG-Ptx levels (>225 IU/ml, suppl. Table 2) than the other age groups (Fig. 4a). Moreover, adolescents, adults and (pre-) elderly showed highest peak responses after pertussis infection for IgG-FHA (>300 IU/ml, suppl. Table 2) than the under-fours, (displaying markedly low anti-FHA responses) and schoolchildren. A reciprocal pattern was observed for IgG-Prn peak responses, which were highest in the three youngest age groups (>100 IU/ml, suppl. Table 2). A prominent age group effect for IgG responses was exhibited by the IgG-Fim2/3 response; (pre-) elderly showed very low IgG-Fim2/3 peak levels (<6 AU/ml), being approximately 5–32-fold lower than the other age groups (Fig. 4a, Suppl. Table 2). Of all age groups, the under-fours showed highest year 3 decay factors for IgG response to 3 out of 4 *B. pertussis* antigens.

IgA responses to all *B. pertussis* antigens were more prominently affected by age group factors than IgG responses (Fig. 4b, Suppl. Table 2). With advancing maturity of the age groups higher medians of predicted IgA levels were found at all time points for vaccine antigens Ptx, FHA and Prn. IgA-Ptx and IgA-FHA peak levels for (pre-) elderly were 19 and 76 fold higher than for the under-fours, respectively. A similar group pattern was observed for IgA-OMV, except that adults displayed higher IgA-OMV responses than the (pre-) elderly did. The under-fours displayed virtually no IgA response to OMV antigens after pertussis infection, reminiscent of the absence of an IgA response to Ptx, FHA, Prn in this age group, With increasing age, higher ‘early’ and late IgA-Fim2/3 levels were observed, except for (pre-) elderly who rather had flat responses at intermediate levels. Noticeably, the under-fours did mount an IgA response to Fim2/3.

### Dissecting *B. pertussis* specific IgG responses after clinical infection at the subclass level: the impact of age group factors

Next, we analysed the IgG response to *B. pertussis* at the level of the IgG1, IgG2, IgG3 and IgG4 subclasses. Subclass dominance, peak response and waning dynamics were identified and compared between age groups. Generally, IgG1 was the prevailing subclass compared to the other three IgG subclasses, as was evidenced by highest fluorescence intensity values. IgG1 levels for all antigens showed similar dynamics as the total IgG levels. (Fig. 5a). Yet other IgG subclasses also contributed to the response.

The anti-OMV response was the only non-IgG1 dominated response, the relative IgG subclass contribution in the total dataset being 44.5% for IgG3, 37% for IgG2, 18% for IgG1, and 0.5% for IgG4. Except the under-fours, all age groups displayed an anti-OMV response. With advancing maturity of the age groups higher IgG2-OMV medians were calculated throughout the entire follow up period. Similarly, IgG3-OMV responses were lowest in under-fours and schoolchildren followed by (pre-) elderly and adults. However highest OMV levels were reached in adolescents (Fig. 5b,c).

Elevated early IgG3 levels were also seen against the *B. pertussis* vaccine antigens Ptx, FHA and Prn, especially in the younger age groups, although low compared to their IgG1 counterparts or to IgG3-OMV levels. IgG3 peak levels in these age groups showed a faster decline than the *B. pertussis* specific IgG decline. For example the week 10 waning factor was 3.9 and 1.8 for IgG3-Ptx levels and 1.7 and 1.4 for IgG-Ptx levels in schoolchildren and adolescents respectively. Waning factors of IgG3-FHA and IgG3-Prn were likewise higher compared to the waning factors of IgG-FHA and IgG-Prn in these age groups (Suppl. Table 2). In under-fours, of whom the majority had received a Fim2/3 containing aP vaccine (Pediacel, Sanofi) in infancy, higher IgG3-Fim2/3 levels were raised than in the other age groups during the first 20 weeks after *B. pertussis* exposure. The day 28 predicted median level of IgG-Fim2/3 for under-fours was 11.7 AU/ml, which declined to 7.6 AU/ml and 0.04 AU/ml at 10 weeks and 2 years, respectively.

IgG4 levels, both at early and later time points after *B. pertussis* exposure, were low in all age groups not vaccinated with aP. Against this background, the under-fours and schoolchildren, being either aP primed and/or aP boosted showed relatively high IgG4 peak levels for the vaccine antigens Ptx (10.9 and 4.4 AU/ml), FHA (17.0 and 43.3 AU/ml) and Prn (25.4 and 5.5 AU/ml). Waning of these levels was fast, after 1 year their predicted IgG4 medians to *B. pertussis* antigens were comparable to the one year medians of the other age groups (Fig. 5d).

### Evidence for reciprocal IgG3 and IgG4 responsiveness related to vaccination history

The modelled data suggested an association between the highest predicted levels for the IgG4 component in the overall anti-*B. pertussis* response with the aP primed cohort of under-fours, while in this age cohort the other minor component in the overall response, IgG3, seemed to wane most prominently. In the cohort of schoolchildren, a small subgroup received aP vaccinations during infancy, while the majority was wP primed, creating the opportunity to investigate the effect of primary vaccination on IgG3 versus IgG4 responsiveness to *B. pertussis* antigens within the same age group. Notably, all schoolchildren received an aP pre-school booster. We dissected unmodelled data for the IgG3 and IgG4 subclasses in the early and late phase of the induced response in sub-groups of schoolchildren with a history of aP or wP priming and compared these responses to the unmodelled data of aP primed under-fours and wP primed adolescents (both groups not having received a pre-school aP booster) (Fig. 6). wP primed and aP primed subjects displayed opposing trends in IgG3 and IgG4 responsiveness. In the early phase, aP primed schoolchildren had lower geomean IgG3 levels and higher geomean IgG4 levels against all antigens than wP primed schoolchildren and adolescents, which was significant for IgG3-Ptx. Adolescents displayed significantly lower IgG4 responses against Ptx, FHA and Prn in the early phase, and significantly higher IgG3-OMV responses both early and late after *B. pertussis* exposure than children having received aP vaccinations (Fig. 6). In the early phase, the (average) relative IgG3 and IgG4 subclass contribution against Ptx was 0.8% and 18.3% for aP primed schoolchildren and 5.6% and 0.1% for adolescents respectively.

## Discussion

Our study is the first to have applied a biexponential decay model to a high-dimensional dataset of *B. pertussis* specific Ig responses obtained from a large cohort of (ex) symptomatic pertussis cases. This greatly facilitated the comparison of early peak levels, decay patterns, maintenance and isotype or IgG subclass usage of Ig responses of various specificities after a clinical pertussis infection. Subsequently important differences were revealed in key features of responses within or between age groups.

First, of all Ig-antigen decay patterns, most extended and steep waning was seen for IgG and IgA to Ptx, when age groups were combined. Waning of IgA levels was steeper than the waning of corresponding IgG levels to Ptx in all age groups, to Prn in all age groups except the (pre-) elderly and to FHA and Fim2/3 in the youngest three age groups. The relatively strong decline of IgG-Ptx, in line with earlier studies^26^,^32^,^38^ could be a possible marker of frailty of the human humoral response to *B. pertussis*, since high levels of IgG-Ptx may prevent symptoms of pertussis^16^. Whether the fast decline of *B. pertussis* specific IgA-levels, consistent with the APERT study^39^ and a study by May *et al*.^40^ also signifies frailty is still unknown. High mucosal IgA levels against adhesins Fim2/3 and FHA may prevent colonization^41^. Though in general serum IgA can trigger effector functions that may extinguish bacteria^18^, a role in protection against *B. pertussis* has not been determined yet. Levels of IgG-Prn in our study showed a more protracted decrease, which could reflect the strong immunogenicity of Prn, also implied by highest IgG-Prn levels in the aP boosted schoolchildren and indirectly suggested by the emergence of Prn deficient strains^42^,^43^. Unlike the study by Berbers *et al*. where a monophasic mathematical model was used to describe pertussis IgG decay after infection or vaccination^32^, we do not see a greater variability in the IgG-FHA and IgG-Prn peak levels than in IgG-Ptx peak levels. Highest variability in our study was found in the Ig response to Fim2/3, both in peak levels as well as when comparing Ig waning patterns among age groups. Notably, of all age groups, under-fours had highest year 3 decay factors for IgG to 3 out of 4 *B. pertussis* antigens. This indicates that in this youngest age group maintenance of serological protection is perhaps most limited, necessitating the use of booster vaccinations at the pre-school age.

Second, IgG1 was the dominant IgG subclass elicited and maintained against most antigens in the *B. pertussis* specific response, likely contributing to effective immunity against *B. pertussis* being one of the complement activating IgG subclasses. Yet, as a clear exception, the response against OMV was dominated by IgG3 (44.5%) and IgG2 (37%). This is in line with a recent study that compared humoral responses in mice induced by various pertussis vaccines including an OMV vaccine and pertussis infection; pertussis infected mice had a similar broad IgG subclass response against OMV^36^. OMVs contain a considerable amount of lipo-oligosaccharide (LOS, a low molecular weight form of lipopolysaccharide; LPS), a known agonist for TLR4. The activation of both TLR4 and the B-cell receptor may lead to T-cell independent anti-LPS IgG2 production^44^,^45^. To our knowledge this is the first study to characterise human IgG subclass responses against (*B. pertussis* specific) OMV. The comparatively high proportion of IgG3 in the OMV specific IgG response is remarkable knowing that IgG3 dominated responses are rarely seen^21^. Since of all IgG subclasses, IgG3 is the strongest complement activator, it could be argued that if OMV based vaccines were to be used, they would provoke not only a broad subclass response but also a powerful one.

Third, several age group dependent features of serological responsiveness to the *B. pertussis* infection were identified, some related to the vaccine regimen changes that were implemented over the last decades in the Netherlands. One example are the higher medians for IgA to Ptx, FHA and Prn at all calculated time points in the response achieved with increasing age. This impact of age on pertussis specific IgA levels has been described before, with the observation that little or no response of IgA-Ptx was found in children <12 years old^40^,^46^. Similar to our study, Prince *et al*. showed an association between age and IgA-Ptx, but not IgG-Ptx^47^. Here we have shown a stepwise increase of IgA levels specific for multiple antigens with age, an effect we earlier observed in a study where IgA-Ptx was used to help calculate the prevalence of pertussis reinfections^35^. Since *B. pertussis* continues to circulate, the cumulative effect of repeated exposures to *B. pertussis* during a lifetime is most likely the cause of the increasing levels of IgA. However, increasing levels of IgG with age were observed to a (much) lesser extent, in line with our earlier work in a subsection of the SKI cohort^34^. Another explanation for the increase of IgA levels conceivably lies in a shifted balance of IgA production and degradation that arises during aging, implied by the increase of IgA nephropathy (the deposition of IgA antibody in the glomeruli of kidneys) with age^48^. Apart from reinforcing immune responses during infections, IgA under normal conditions has a role as an anti-inflammatory antibody that maintains homeostasis^49^,^50^; hence high IgA levels in elderly do not necessarily signify a higher level of protection against infections. More research on the role of serum IgA in the protection against *B. pertussis* is entailed.

Another example of age group dependency identified by the model are the very low IgG and IgA levels to Fim2/3 observed in the (pre-) elderly. The subjects in this age group did not receive pertussis vaccination in infancy; hence their responses were induced by natural infection only, surely by the proven symptomatic infection central in this study but likely also by (silent) pertussis infections earlier in life. *B. pertussis* can express Fim2, Fim3 or both antigens, and for each of these antigenic shifts have occurred over time^51^,^52^, but natural infection has been shown to be poorly immunogenic for Fim, especially Fim3^26^,^53^,^54^. Fim serotyping of clinical isolates from our SKI-cases (with diagnoses made between 1992 and 2012) is not available, yet it is unlikely that these involved only low immunogenic Fim3 strains. Apparently, life-long exposure to circulating strains in the (pre-) elderly does not generate strong Fim responsiveness *in vitro*, and having received a fimbriae-containing pertussis vaccine seems required, as was earlier suggested^53^. In line with this, a small subgroup of under-fours (n = 4) that were primed with an aP vaccine lacking a Fim2/3 component likewise showed minimal anti Fim2/3 responses after infection, while the under-fours primed in infancy with a Fim2/3 containing aP vaccine (n = 30) showed high anti Fim2/3 responses after infection. Our adults and adolescents having been primed with wP (generated from *B. pertussis* strains producing both Fim2 and Fim3) displayed substantial IgG-Fim2/3 responses after natural infection. Remarkably, while the under-fours elicited almost no IgA responses to all other antigens, a moderate IgA-Fim2/3 peak level was predicted for this age group, that quickly decreased (12-fold by week 20). As anti-fimbriae antibodies have been shown to be important in protection against pertussis^9^,^10^,^55^, the absence of these antibodies in both non-vaccinated (pre-) elderly and infants vaccinated with a non-fimbriae containing aP might indicate a higher risk of a recurrent pertussis infection for these age groups than for age groups vaccinated in infancy with a Fim2/3 containing pertussis vaccine. Implementing, where applicable, vaccination with a Fim2/3 containing vaccine both in primary series and in any booster situation, could perhaps partly improve the current suboptimal duration of protection after aP vaccination^4^,^5^. However, as indicated below, further measures will be needed to optimize pertussis vaccination.

As a final example of an age group effect, the vaccination history impacted the IgG3 and IgG4 subclass responsiveness to aP vaccine antigens in the three younger age cohorts. Considerable IgG3 and IgG4 peak levels and steep waning factors were predicted by our biexponential model for Ptx, FHA and Prn in the three younger age groups. Remarkably, the unmodelled data showed a reciprocal trend in responsiveness of IgG3 and the allergy associated IgG4 subclass in aP primed children and wP primed children and adolescents. Already in 1989, an increased IgG3-Ptx response was found in Sweden in naturally infected pertussis patients and not in aP vaccinated individuals^56^. An Italian study similarly found practically undetectable levels of IgG3 produced by children vaccinated with aP while high responses were found in children with whooping cough previously vaccinated with a wP or a DT only vaccine^57^. On the other hand, Hendrikx *et al*. found hardly any IgG3 responses in either healthy vaccinated or naturally infected children but did find increasing IgG4 levels in aP primed children with accumulating numbers of aP vaccinations^58^. Consistent with our study, an acellular pertussis toxoid vaccine was found to induce both IgG1 and IgG4 antibodies^56^. IgG4 antibodies are usually seen following prolonged immunization, and are associated with Th-2 skewing of the immune system. Vaccination with aP vaccines has been associated with Th-2 type T cell responses^59^. Switching to IgG4 could be regulated by IL-10 and other anti-inflammatory cytokines signifying tolerance induction^21^,^25^. Our data illustrate that the association between aP vaccination and IgG4 responsiveness remains imprinted in the immune response, even when recalled by natural infection. This information is important in the current discussion how to improve current (aP) vaccines or to develop third generation novel vaccines, steering the immune response towards a more protective Th-1 dominated type of immune response and with a more favourable (thus higher) IgG3/IgG4 ratio.

Some factors inherent to our study population or methodology may warrant caution in the interpretation of the data. While studying immune responses to a known natural *B. pertussis* infection in a large human cohort, earlier unknown exposures to live *B. pertussis* throughout life may have influenced the results. Although all our cases had a symptomatic pertussis infection, most had vaccinations before, but some received vaccinations after the infection. Sixty-two samples (18.7%) were of young patients, whose symptomatic infection was followed by one or more childhood pertussis vaccinations. We found that Ig levels obtained by the model when these specific cases were interpreted separately did not show great differences. Yet, we tend to agree that the way the immune response is primed early in life, either by a vaccination (aP or wP) or a natural infection, impacts responsiveness later in life^60^, and that this needs further investigation. Also, we modelled the dynamics of responses in our (ex-) cases at the level of five age cohorts defined by clear differences in vaccination background based on age, yet our two youngest age cohorts encompassed within-cohort heterogeneity in vaccine types used (Fig. 1 and Supplementary Table 1). Differences in antigen composition in within-cohort used vaccines may have caused within-cohort variation in specificities, and, as shown in the sub-analysis of aP versus wP primed subgroups within the schoolchildrens’ age cohort, vaccine type impacted IgG3 and IgG4 responsiveness. Therefore modelled data for the more heterogeneous younger age cohorts should be interpreted with caution. While in general a Multiplex Immuno-assay has higher sensitivity than classical ELISAs^37^, for several Ig-antigen combinations multiple samples had concentrations below the lower limit of detection. In order to still be able to evaluate these data we did not remove these points from the datasets but instead assigned them a model-based concentration in the order of this lower limit, to indicate their low responsiveness. For these datasets, modelled parameters and trends were interpreted with caution.

In conclusion, the biexponential decay model enabled us to effectively capture critical features in the long-term kinetics of concomitant Ig responses ongoing after a symptomatic *B. pertussis* infection. The merit of this work is that it illustrates that across age groups, immunity to *B. pertussis* and waning thereof, involve diverging responses of intrinsically different Ig subtypes, which are dictated by immunogenicity profiles of antigens, and cohort factors such as age and vaccination history. Waning of *B. pertussis* specific immunity requires urgent attention with the growing awareness that pertussis affects all age groups and that current acellular vaccines fail to provide long lasting immunity. As novel intervention strategies in different age groups are becoming realistic scenarios, detailed benchmarking of *B. pertussis* immune responsiveness throughout life is of utmost importance.

## Additional Information

**How to cite this article**: van Twillert, I. *et al*. Impact of age and vaccination history on long-term serological responses after symptomatic *B. pertussis* infection, a high dimensional data analysis. *Sci. Rep.* **7**, 40328; doi: 10.1038/srep40328 (2017).

**Publisher's note:** Springer Nature remains neutral with regard to jurisdictional claims in published maps and institutional affiliations.

## Supplementary Material

Supplementary Information

## Figures and Tables

**Table 1:** IgG and IgA levels (50^th^ percentiles) of all SKI cases at day 28, week 10, year 2 and year 3, based on the model and corresponding waning factors against *B. pertussis* antigens.

IgG	peak [Ig]t = d28	[Ig]t = wk10	[Ig]t = 2y	[Ig]t = 3y	Week 10 waning factor*	Year 2 waning factor*	Year 3 waning factor*	2.5–97.5^th^ percentile range of [Ig]t = d28
Ptx	209.7	122.7	29.5	25.3	**1.7**	**7.1**	**8.3**	29.1–1314.1
FHA	330.0	251.8	71.9	58.9	**1.3**	**4.6**	**5.6**	63.7–2031.7
Prn	88.8	76.2	45.0	37.2	**1.2**	**2.0**	**2.4**	11.6–755.5
Fim2/3	47.7	23.3	9.1	6.2	**2.0**	**5.2**	**7.7**	4.1–3052.2
OMV	ND	ND	ND	ND	ND	ND	ND	ND
**IgA**	**peak [Ig]t** = **d28**	**[Ig]t** = **wk10**	**[Ig]t** = **2y**	**[Ig]t** = **3y**	**ratio d28/wk10**	**ratio d28/2y**	**ratio d28/3y**	**2.5–97.5**^**th**^ **percentile range of [Ig]t** = **d28**
Ptx	12.8	4.3	1.5	1.4	**2.9**	**8.7**	**8.9**	0.8–176.2
FHA	47.1	23.1	9.1	7.0	**2.0**	**5.2**	**6.8**	4.0–1047.3
Prn	29.9	12.4	6.6	6.1	**2.4**	**4.5**	**4.9**	2.0–1346.3
Fim2/3	103.1	40.0	16.0	12.4	**2.6**	**6.5**	**8.3**	7.0–3932.3
OMV	133.7	59.4	29.2	28.8	**2.3**	**4.6**	**4.6**	10.9–1558.4

^*^As described in Methods, ND = Not Determined.
