# Supplementary material for: Impact of age and vaccination history on long-term serological responses after symptomatic B. pertussis infection, a high dimensional data analysis
Source: Sci Rep. 2017 Jan 16;7:40328. doi: 10.1038/srep40328 (PMC5238437; doi:10.1038/srep40328)
Supplement: Supplementary Information [file srep40328-s1.pdf]

Impact of age and vaccination history on long-term serological responses after symptomatic *B. pertussis* infection, a high dimensional data analysis

Inonge van Twillert[1], Axel A. Bonačić Marinović[1], Betsy Kuipers[1], Jacqueline A. M. van Gaans-van den Brink[1], Elisabeth A. M. Sanders[1,2], Cécile A.C.M. van Els[1]

[1]Centre for Infectious Disease Control, National Institute for Public Health and the Environment (RIVM), Bilthoven, the Netherlands, [2] Department of Immunology and Infectious Diseases, Wilhelmina Childrens Hospital, University Medical Center Utrecht, Utrecht, the Netherlands

Supplementary table 1: Vaccination history of SKI schoolchildren

Patient code	Age at bloodsampling	Vaccination or Infection last?	Primary vacc. Series	Product Primary series*	pre-school aP booster?	Product Pre-school aP booster**
CI67399	11.6	Inf	No vacc.	N.A.	No	N.A.
CI67099	11.3	Inf	No vacc.	N.A.	No	N.A.
GH61999	11.9	Inf	No vacc.	N.A.	No	N.A.
GH62099	13.1	Inf	No vacc.	N.A.	No	N.A.
CI70299	9.1	Inf	No vacc.	N.A.	No	N.A.
CI63999	5.5	Inf	No vacc.	N.A.	No	N.A.
HU07801	4.7	Inf	No vacc.	N.A.	No	N.A.
HU06101	8.2	Inf	No vacc.	N.A.	No	N.A.
CI71299	7.0	Inf	wP	NVI, only pertussis component at 2 months	No	N.A.
CI64099	5.7	Vacc	wP	NVI	No	N.A.
CI67599	12.1	Vacc	wP	NVI	yes	stand alone aP (GSK)
CI67199	11.8	Vacc	wP	NVI	yes	stand alone aP (GSK)
CI67499	12.1	Vacc	wP	NVI	yes	stand alone aP (GSK)
CI66699	11.7	Vacc	wP	NVI	yes	stand alone aP (GSK)
CI66999	11.2	Vacc	wP	NVI	yes	stand alone aP (GSK)
CI66799	10.7	Vacc	wP	NVI	yes	stand alone aP (GSK)
CI66599	11.2	Vacc	wP	NVI	yes	stand alone aP (GSK)
CI70899	10.0	Vacc	wP	NVI	yes	stand alone aP (GSK)
GH62999	10.3	Vacc	wP	NVI	yes	stand alone aP (GSK)
CI64999	10.1	Vacc	wP	NVI	yes	stand alone aP (GSK)
GH60199	9.4	Vacc	wP	NVI	yes	stand alone aP (GSK)
CI70799	9.9	Vacc	wP	NVI	yes	stand alone aP (GSK)
CI70699	9.5	Vacc	wP	NVI	yes	stand alone aP (GSK)
GH60599	9.8	Vacc	wP	NVI	yes	stand alone aP (GSK)
HU63399	9.0	Vacc	wP	NVI	yes	stand alone aP (GSK)
CI64699	9.5	Vacc	wP	NVI	yes	stand alone aP (GSK)
CI64599	8.8	Vacc	wP	NVI	yes	stand alone aP (GSK)
GH62399	8.6	Vacc	wP	NVI	yes	stand alone aP (GSK)
CI72399	8.1	Vacc	wP	NVI	yes	Triaxis (SP)
CI71199	8.1	Vacc	wP	NVI	yes	stand alone aP (GSK)
CI64199	8.0	Vacc	wP	NVI	yes	stand alone aP (GSK)
CI64499	7.9	Vacc	wP	NVI	yes	stand alone aP (GSK)
CI71799	7.9	Vacc	wP	NVI	yes	Triaxis (SP)
CI64899	8.3	Vacc	wP	NVI	yes	stand alone aP (GSK)
CI70599	7.7	Vacc	wP	NVI	yes	stand alone aP (GSK)
CI70499	7.1	Vacc	wP	NVI	yes	stand alone aP (GSK)
HU61799	7.7	Vacc	wP	NVI	yes	stand alone aP (GSK)
CI72099	6.9	Vacc	wP	NVI	yes	Infanrix-IPV (GSK)
CI65099	7.0	Vacc	wP	NVI	yes	Triaxis (SP)
CI70199	6.7	Vacc	wP	NVI	yes	Triaxis (SP)
GH62499	6.7	Vacc	wP	NVI	yes	Triaxis (SP)
CI70399	6.4	Vacc	wP	NVI	yes	Triaxis (SP)
CI64799	6.4	Vacc	wP	NVI	yes	unknown
CI64299	6.0	Vacc	wP	NVI	yes	Infanrix-IPV (GSK)
CI63699	5.7	Vacc	wP	NVI	yes	Infanrix-IPV (GSK)
HU05902	11.4	Inf	wP	NVI	yes	stand alone aP (GSK)
HU05502	10.2	Inf	wP	NVI	yes	stand alone aP (GSK)
HU05802	10.4	Inf	wP	NVI	yes	stand alone aP (GSK)
HU06902	11.3	Inf	wP	NVI	yes	stand alone aP (GSK)
HU06502	10.1	Inf	wP	NVI	yes	stand alone aP (GSK)
HU05702	12.9	Inf	wP	NVI	yes	stand alone aP (GSK)
HU06802	12.0	Inf	wP	NVI	yes	stand alone aP (GSK)
HU52699	9.7	Inf	wP	NVI	yes	stand alone aP (GSK)
HU54899	10.2	Inf	wP	NVI	yes	stand alone aP (GSK)
HU54999	10.2	Inf	wP	NVI	yes	stand alone aP (GSK)
HU54199	10.4	Inf	wP	NVI	yes	stand alone aP (GSK)
HU54299	8.0	Inf	wP	NVI	yes	stand alone aP (GSK)

HU05901	8.2	Inf	wP	NVI	yes	stand alone aP (GSK)
HU08201	9.7	Inf	wP	NVI	yes	stand alone aP (GSK)
HU07101	7.6	Inf	wP	NVI	yes	stand alone aP (GSK)
HU54099	9.1	Inf	wP	NVI	yes	stand alone aP (GSK)
HU07301	7.5	Inf	wP	NVI	yes	stand alone aP (GSK)
HU07201	8.4	Inf	wP	NVI	yes	stand alone aP (GSK)
HU06901	8.4	Inf	wP	NVI	yes	stand alone aP (GSK)
HU08001	8.3	Inf	wP	NVI	yes	Triaxis (SP)
SP51999	10.3	Inf	wP	NVI	yes	stand alone aP (GSK)
HU05801	7.3	Inf	wP	NVI	yes	stand alone aP (GSK)
HU06601	6.4	Inf	wP	NVI	yes	Triaxis (SP)
HU07601	7.3	Inf	wP	NVI	yes	Triaxis (SP)
HU06401	9.3	Inf	wP	NVI	yes	stand alone aP (GSK)
HU06001	9.7	Inf	wP	NVI	yes	stand alone aP (GSK)
HU05401	9.2	Inf	wP	NVI	yes	stand alone aP (GSK)
HU06501	7.2	Inf	wP	NVI	yes	stand alone aP (GSK)
HU08401	8.4	Inf	wP	NVI	yes	Triaxis (SP)
HU06201	6.4	Inf	wP	NVI	yes	Triaxis (SP)
HU53599	9.5	Inf	wP	NVI	yes	stand alone aP (GSK)
HU53499	5.5	Inf	wP	NVI	yes	Triaxis (SP)
HU06801	9.3	Inf	wP	NVI	yes	stand alone aP (GSK)
HU05301	8.4	Inf	wP	NVI	yes	stand alone aP (GSK)
HU07001	6.9	Inf	wP	NVI	yes	Triaxis (SP)
HU05201	8.6	Inf	wP	NVI	yes	stand alone aP (GSK)
HU05701	10.0	Inf	wP	NVI	yes	stand alone aP (GSK)
HU05501	6.9	Inf	wP	NVI	yes	stand alone aP (GSK)
HU08301	9.8	Inf	wP	NVI	yes	Triaxis (SP)
HU01021	7.9	inf	wP	NVI	yes	Triaxis (SP)
HU07501	8.5	Inf	wP	NVI	yes	stand alone aP (GSK)
HU11101	10.3	Inf	wP	NVI	yes	stand alone aP (GSK)
CI71599	6.4	Vacc	mixed wP/aP	wP mnths 2, 3, 4 (NVI) , mnth11 aP Infanrix-IPV-Hib (GSK)	yes	Triaxis (SP)
CI63799	5.5	Vacc	mixed wP/aP	wP mnths 2, 3, 4 (NVI) , mnth11 aP Infanrix-IPV-Hib (GSK)	yes	Infanrix-IPV (GSK)
HU51099	4.1	Vacc	mixed wP/aP	wP mnths 2, 3, 4 (NVI) , mnth11 aP Infanrix-IPV-Hib (GSK)	yes	Infanrix-IPV (GSK)
GH07901	7.3	Inf	mixed wP/aP	wP mnths 2, 3, 4 (NVI) , mnth11 aP Infanrix-IPV-Hib (GSK)	yes	Triaxis (SP)
HU05101	4.6	Inf	mixed wP/aP	wP mnths 2, 3, 4 (NVI) , mnth11 aP Infanrix-IPV-Hib (GSK)	yes	Triaxis (SP)
CI71499	6.3	Vacc	aP	Infanrix-IPV-Hib (GSK)	yes	Triaxis (SP)
CI58099	4.2	Vacc	aP	Pediacel (SP)	yes	Infanrix-IPV
CI71399	5.6	Vacc	aP	mnths 2,3,4 Infanrix-IPV-Hib (GSK), mnth 11 Pediacel (SP)	yes	Infanrix-IPV (GSK)
CI70999	5.4	Vacc	aP	mnths 2,3,4 Infanrix-IPV-Hib (GSK,) mnth 11 Pediacel (SP)	yes	Infanrix-IPV (GSK)
CI57299	3.8	Vacc	aP	Pediacel (SP)	yes	Infanrix-IPV (GSK)
HU71899	4.3	Inf	aP	Pediacel (SP)	yes	Infanrix-IPV (GSK)
HU71999	4.9	Inf	aP	Pediacel (SP)	yes	Infanrix-IPV (GSK)
HU07401	4.8	Inf	aP	mnth 2,3,4 Infanrix-IPV-Hib (GSK), mnth 11 Pediacel (SP)	yes	Infanrix-IPV (GSK)
HU54399	5.0	Inf	aP	mnth 2,3,4 Infanrix-IPV-Hib (GSK), mnth 11 Pediacel (SP)	yes	Infanrix-IPV (GSK)
HU08501	4.8	Inf	aP	Pediacel (SP)	yes	Infanrix-IPV (GSK)
HU07701	4.2	Inf	aP	Pediacel (SP)	yes	Infanrix-IPV (GSK)

Manufacturers: GSK= GlaxoSmithKline, SP=Sanofi Pasteur, NVI=Netherlands Vaccine Institute (or predecessor)

* Used Primary vaccines and their *B. pertussis* antigen dose: Infanrix-IPV-Hib (GSK): threevalent pertussis vaccine (Ptx: 25 ug, FHA: 25 ug, Prn: 8 ug); Pediacel (SP): five-valent pertussis vaccine (Ptx: 20 ug, FHA: 20 ug, Prn: 3 ug, Fim2/Fim3: 5 ug).

** Used booster vaccines and their *B. pertussis* antigen dose: stand alone aP (GSK): (Ptx: 25 ug, FHA: 25 ug, Prn: 8 ug); Triaxis (SP): five-valent aP booster (Ptx: 2.5 ug, FHA: 5 ug, Prn: 3 ug, Fim2/Fim3: 5 ug); Infanrix-IPV (GSK): threevalent pertussis vaccine (Ptx: 25 ug, FHA: 25 ug, Prn: 8 ug)

Supplementary table 2: Model based IgG, IgA and IgG subclass levels (50th percentiles) of SKI age groups at day 28, week 10, year 2 and year 3, and corresponding waning factors against *B. pertussis* antigens

	Ptx							FHA							Prn						
IgG	[Ig]t=d28	[Ig]t=wk10	[Ig]t=2y	[Ig]t=3y	ratio d28/wk10	ratio d28/2y	ratio d28/3y	[Ig]t=d28	[Ig]t=wk10	[Ig]t=2y	[Ig]t=3y	ratio d28/wk10	ratio d28/2y	ratio d28/3y	[Ig]t=d28	[Ig]t=wk10	[Ig]t=2y	[Ig]t=3y	ratio d28/wk10	ratio d28/2y	ratio d28/3y
All ages	209.7	122.7	29.5	25.3	1.7	7.1	8.3	330.0	251.8	71.9	58.9	1.3	4.6	5.6	88.8	76.2	45.0	37.2	1.2	2.0	2.4
u4s	120.3	58.0	11.9	10.6	2.1	10.1	11.4	91.0	80.4	20.8	10.8	1.1	4.4	8.4	103.2	59.7	14.4	9.9	1.7	7.2	10.4
sch	197.2	114.9	45.9	40.7	1.7	4.3	4.8	233.1	219.0	130.2	106.0	1.1	1.8	2.2	156.9	152.4	95.8	77.9	1.0	1.6	2.0
ado	180.3	131.7	17.4	13.0	1.4	10.3	13.8	482.1	391.5	93.4	70.8	1.2	5.2	6.8	110.5	103.2	51.0	37.8	1.1	2.2	2.9
adu	226.3	173.1	34.4	23.6	1.3	6.6	9.6	302.1	259.3	105.2	85.7	1.2	2.9	3.5	63.0	60.4	34.1	25.2	1.0	1.8	2.5
eld	289.7	201.8	41.3	35.0	1.4	7.0	8.3	481.4	391.3	151.4	126.4	1.2	3.2	3.8	80.3	69.9	27.1	18.2	1.1	3.0	4.4

IgA	peak (d28)	[Ig]t=wk10	[Ig]t=2y	[Ig]t=3y	ratio d28/wk10	ratio d28/2y	ratio d28/3y	peak (d28)	[Ig]t=wk10	[Ig]t=2y	[Ig]t=3y	ratio d28/wk10	ratio d28/2y	ratio d28/3y	peak (d28)	[Ig]t=wk10	[Ig]t=2y	[Ig]t=3y	ratio d28/wk10	ratio d28/2y	ratio d28/3y
All ages	12.8	4.3	1.5	1.4	2.9	8.7	8.9	47.1	23.1	9.1	7.0	2.0	5.2	6.8	29.9	12.4	6.6	6.1	2.4	4.5	4.9
u4s	1.7	0.6	0.3	0.3	3.0	5.6	5.6	8.1	1.9	0.1	0.1	4.3	56.8	72.4	1.9	0.8	0.6	0.6	2.4	3.0	3.0
sch	5.7	2.9	1.3	1.3	2.0	4.3	4.3	37.7	11.4	4.1	3.9	3.3	9.1	9.7	30.9	9.1	4.2	4.0	3.4	7.3	7.7
ado	10.1	4.7	1.1	1.1	2.1	9.0	9.5	68.0	44.2	9.8	7.8	1.5	6.9	8.7	47.8	20.3	9.7	8.5	2.4	4.9	5.6
adu	14.6	9.2	3.5	3.3	1.6	4.2	4.5	63.4	60.5	30.7	22.6	1.0	2.1	2.8	47.5	36.3	21.1	18.1	1.3	2.3	2.6
eld	33.1	17.6	8.6	8.1	1.9	3.8	4.1	234.3	202.4	98.0	78.1	1.2	2.4	3.0	101.7	97.6	56.8	44.3	1.0	1.8	2.3

IgG1	peak (d28)	[Ig]t=wk10	[Ig]t=2y	[Ig]t=3y	ratio d28/wk10	ratio d28/2y	ratio d28/3y	peak (d28)	[Ig]t=wk10	[Ig]t=2y	[Ig]t=3y	ratio d28/wk10	ratio d28/2y	ratio d28/3y	peak (d28)	[Ig]t=wk10	[Ig]t=2y	[Ig]t=3y	ratio d28/wk10	ratio d28/2y	ratio d28/3y
All ages	162.9	95.5	26.5	22.9	1.7	6.1	7.1	226.5	159.0	81.8	71.1	1.4	2.8	3.2	63.1	60.3	36.8	29.6	1.0	1.7	2.1
u4s	97.3	54.4	8.0	7.1	1.8	12.2	13.7	71.0	61.5	15.7	8.2	1.2	4.5	8.7	45.9	40.3	10.4	5.5	1.1	4.4	8.4
sch	188.9	105.9	48.6	43.6	1.8	3.9	4.3	169.5	163.1	111.8	93.9	1.0	1.5	1.8	109.6	105.2	65.2	51.1	1.0	1.7	2.1
ado	121.3	88.0	17.0	13.5	1.4	7.1	9.0	321.1	271.7	90.4	72.1	1.2	3.6	4.5	68.7	67.2	41.8	34.6	1.0	1.6	2.0
adu	159.9	132.3	30.3	20.4	1.2	5.3	7.9	202.7	182.1	81.7	65.4	1.1	2.5	3.1	45.7	43.8	26.2	20.9	1.0	1.7	2.2
eld	236.9	169.7	37.3	31.4	1.4	6.4	7.6	279.6	238.5	107.4	88.1	1.2	2.6	3.2	48.0	44.4	17.8	11.9	1.1	2.7	4.0

IgG2	peak (d28)	[Ig]t=wk10	[Ig]t=2y	[Ig]t=3y	ratio d28/wk10	ratio d28/2y	ratio d28/3y	peak (d28)	[Ig]t=wk10	[Ig]t=2y	[Ig]t=3y	ratio d28/wk10	ratio d28/2y	ratio d28/3y	peak (d28)	[Ig]t=wk10	[Ig]t=2y	[Ig]t=3y	ratio d28/wk10	ratio d28/2y	ratio d28/3y
All ages	0.63	0.39	0.19	0.15	1.6	3.3	4.3	2.83	2.52	1.30	0.98	1.1	2.2	2.9	1.25	0.72	0.53	0.50	1.7	2.3	2.5
u4s	0.97	0.79	0.12	0.05	1.2	8.3	19.9	2.62	2.29	0.64	0.37	1.1	4.1	7.1	0.85	0.79	0.75	0.73	1.1	1.1	1.2
sch	0.84	0.52	0.23	0.17	1.6	3.7	5.0	4.54	3.01	0.81	0.60	1.5	5.6	7.5	3.35	1.17	0.57	0.56	2.9	5.9	6.0
ado	0.25	0.24	0.16	0.13	1.0	1.5	1.9	2.06	1.97	1.20	0.95	1.0	1.7	2.2	1.10	0.70	0.52	0.46	1.6	2.1	2.4
adu	0.53	0.38	0.09	0.07	1.4	5.7	7.9	2.86	2.30	1.44	1.20	1.2	2.0	2.4	0.76	0.45	0.38	0.38	1.7	2.0	2.0
eld	0.50	0.30	0.07	0.06	1.7	7.2	7.8	1.73	1.50	0.61	0.49	1.2	2.8	3.5	0.57	0.23	0.19	0.19	2.4	3.1	3.1

IgG3	peak (d28)	[Ig]t=wk10	[Ig]t=2y	[Ig]t=3y	ratio d28/wk10	ratio d28/2y	ratio d28/3y	peak (d28)	[Ig]t=wk10	[Ig]t=2y	[Ig]t=3y	ratio d28/wk10	ratio d28/2y	ratio d28/3y	peak (d28)	[Ig]t=wk10	[Ig]t=2y	[Ig]t=3y	ratio d28/wk10	ratio d28/2y	ratio d28/3y
All ages	0.68	0.44	0.14	0.09	1.5	4.7	7.8	1.43	1.03	0.58	0.46	1.4	2.4	3.1	1.36	0.57	0.41	0.41	2.4	3.3	3.3
u4s	1.16	0.53	0.02	0.01	2.2	50.1	106.4	2.09	1.63	0.09	0.02	1.3	23.6	113.7	1.42	0.73	0.02	0.01	1.9	73.9	203.8
sch	2.60	0.67	0.14	0.13	3.9	19.3	19.9	2.57	1.53	0.94	0.79	1.7	2.8	3.2	2.79	0.93	0.60	0.60	3.0	4.7	4.7
ado	1.24	0.69	0.17	0.10	1.8	7.4	12.4	1.92	1.01	0.55	0.48	1.9	3.5	4.0	2.07	1.16	0.72	0.61	1.8	2.9	3.4
adu	0.31	0.28	0.10	0.06	1.1	3.2	5.2	0.76	0.73	0.43	0.34	1.0	1.8	2.2	0.95	0.37	0.27	0.27	2.5	3.5	3.5
eld	0.37	0.31	0.04	0.02	1.2	8.7	21.1	0.94	0.71	0.35	0.26	1.3	2.7	3.6	1.18	0.48	0.31	0.31	2.5	3.8	3.8

IgG4	peak (d28)	[Ig]t=wk10	[Ig]t=2y	[Ig]t=3y	ratio d28/wk10	ratio d28/2y	ratio d28/3y	peak (d28)	[Ig]t=wk10	[Ig]t=2y	[Ig]t=3y	ratio d28/wk10	ratio d28/2y	ratio d28/3y	peak (d28)	[Ig]t=wk10	[Ig]t=2y	[Ig]t=3y	ratio d28/wk10	ratio d28/2y	ratio d28/3y
All ages	0.52	0.22	0.09	0.07	2.4	6.0	7.9	4.56	0.45	0.21	0.21	10.2	21.4	22.2	0.28	0.10	0.08	0.08	2.7	3.6	3.7
u4s	10.90	6.41	0.01	0.00	1.7	1388.0	36551.9	16.95	10.72	0.05	0.00	1.6	311.8	5266.1	25.43	3.70	0.07	0.06	6.9	356.2	400.4
sch	4.44	0.64	0.17	0.13	6.9	26.0	34.3	43.26	3.83	0.45	0.36	11.3	96.3	121.1	5.52	0.57	0.23	0.19	9.7	23.9	29.0
ado	0.05	0.05	0.03	0.03	1.0	1.6	2.0	0.40	0.23	0.16	0.15	1.8	2.5	2.7	0.07	0.05	0.04	0.04	1.6	1.8	1.8
adu	0.39	0.13	0.06	0.05	3.0	6.4	7.2	0.83	0.35	0.25	0.25	2.3	3.3	3.3	0.07	0.05	0.03	0.03	1.4	2.0	2.1
eld	0.30	0.26	0.06	0.03	1.2	4.7	8.7	0.14	0.11	0.08	0.07	1.2	1.8	2.1	0.05	0.04	0.02	0.01	1.2	3.0	4.1

	Fim2/3							OMV						
**IgG**	**[Ig]t=d28**	**[Ig]t=wk10**	**[Ig]t=2y**	**[Ig]t=3y**	**ratio d28/wk10**	**ratio d28/2y**	**ratio d28/3y**	**[Ig]t=d28**	**[Ig]t=wk10**	**[Ig]t=2y**	**[Ig]t=3y**	**ratio d28/wk10**	**ratio d28/2y**	**ratio d28/3y**
**All ages**	47.7	23.3	9.1	6.2	**2.0**	**5.2**	**7.7**	ND	ND	ND	ND	**ND**	**ND**	**ND**
**u4s**	105.0	75.5	2.2	0.3	**1.4**	**48.6**	**345.7**	ND	ND	ND	ND	**ND**	**ND**	**ND**
**sch**	33.5	15.4	5.3	3.5	**2.2**	**6.4**	**9.5**	ND	ND	ND	ND	**ND**	**ND**	**ND**
**ado**	159.5	62.3	23.8	20.6	**2.6**	**6.7**	**7.7**	ND	ND	ND	ND	**ND**	**ND**	**ND**
**adu**	149.3	81.0	44.2	36.6	**1.8**	**3.4**	**4.1**	ND	ND	ND	ND	**ND**	**ND**	**ND**
**eld**	5.3	0.6	0.3	0.3	**9.1**	**19.7**	**20.2**	ND	ND	ND	ND	**ND**	**ND**	**ND**

**IgA**	**peak (d28)**	**[Ig]t=wk10**	**[Ig]t=2y**	**[Ig]t=3y**	**ratio d28/wk10**	**ratio d28/2y**	**ratio d28/3y**	**peak (d28)**	**[Ig]t=wk10**	**[Ig]t=2y**	**[Ig]t=3y**	**ratio d28/wk10**	**ratio d28/2y**	**ratio d28/3y**
**All ages**	103.1	40.0	16.0	12.4	**2.6**	**6.5**	**8.3**	133.7	59.4	29.2	28.8	**2.3**	**4.6**	**4.6**
**u4s**	103.8	38.9	0.9	0.8	**2.7**	**113.1**	**131.9**	8.9	8.9	8.9	8.8	**1.0**	**1.0**	**1.0**
**sch**	58.2	19.2	6.4	4.7	**3.0**	**9.1**	**12.3**	61.6	36.6	17.9	17.6	**1.7**	**3.4**	**3.5**
**ado**	359.3	124.1	29.2	23.2	**2.9**	**12.3**	**15.5**	204.3	122.2	43.7	40.5	**1.7**	**4.7**	**5.0**
**adu**	247.2	231.3	122.7	89.0	**1.1**	**2.0**	**2.8**	217.7	180.4	87.4	74.9	**1.2**	**2.5**	**2.9**
**eld**	39.0	19.4	15.7	15.7	**2.0**	**2.5**	**2.5**	271.5	190.4	118.8	113.4	**1.4**	**2.3**	**2.4**

**IgG1**	**peak (d28)**	**[Ig]t=wk10**	**[Ig]t=2y**	**[Ig]t=3y**	**ratio d28/wk10**	**ratio d28/2y**	**ratio d28/3y**	**peak (d28)**	**[Ig]t=wk10**	**[Ig]t=2y**	**[Ig]t=3y**	**ratio d28/wk10**	**ratio d28/2y**	**ratio d28/3y**
**All ages**	136.1	55.9	27.7	22.8	**2.4**	**4.9**	**6.0**	49.9	27.2	18.1	18.1	**1.8**	**2.8**	**2.8**
**u4s**	331.3	245.3	7.0	1.0	**1.4**	**47.1**	**320.7**	6.4	6.4	6.3	6.2	**1.0**	**1.0**	**1.0**
**sch**	95.4	42.6	18.8	15.3	**2.2**	**5.1**	**6.2**	38.5	25.8	13.8	13.5	**1.5**	**2.8**	**2.9**
**ado**	497.5	209.4	79.6	71.0	**2.4**	**6.3**	**7.0**	127.7	80.2	30.0	28.2	**1.6**	**4.3**	**4.5**
**adu**	291.1	275.9	154.7	120.1	**1.1**	**1.9**	**2.4**	70.2	57.3	31.2	28.3	**1.2**	**2.3**	**2.5**
**eld**	11.8	2.8	1.5	1.5	**4.2**	**7.9**	**7.9**	40.1	29.1	19.1	18.5	**1.4**	**2.1**	**2.2**

**IgG2**	**peak (d28)**	**[Ig]t=wk10**	**[Ig]t=2y**	**[Ig]t=3y**	**ratio d28/wk10**	**ratio d28/2y**	**ratio d28/3y**	**peak (d28)**	**[Ig]t=wk10**	**[Ig]t=2y**	**[Ig]t=3y**	**ratio d28/wk10**	**ratio d28/2y**	**ratio d28/3y**
**All ages**	3.83	1.62	0.77	0.66	**2.4**	**5.0**	**5.8**	37.46	19.94	12.56	11.24	**1.9**	**3.0**	**3.3**
**u4s**	6.35	4.49	0.53	0.23	**1.4**	**12.0**	**28.1**	2.42	2.41	2.38	2.34	**1.0**	**1.0**	**1.0**
**sch**	4.69	0.83	0.45	0.44	**5.7**	**10.5**	**10.7**	29.66	12.70	4.58	4.44	**2.3**	**6.5**	**6.7**
**ado**	13.76	7.81	2.86	2.15	**1.8**	**4.8**	**6.4**	26.94	25.73	18.38	15.98	**1.0**	**1.5**	**1.7**
**adu**	6.34	4.54	2.19	1.71	**1.4**	**2.9**	**3.7**	115.72	107.48	66.34	53.40	**1.1**	**1.7**	**2.2**
**eld**	0.24	0.20	0.19	0.19	**1.2**	**1.2**	**1.2**	136.85	133.38	95.34	80.10	**1.0**	**1.4**	**1.7**

**IgG3**	**peak (d28)**	**[Ig]t=wk10**	**[Ig]t=2y**	**[Ig]t=3y**	**ratio d28/wk10**	**ratio d28/2y**	**ratio d28/3y**	**peak (d28)**	**[Ig]t=wk10**	**[Ig]t=2y**	**[Ig]t=3y**	**ratio d28/wk10**	**ratio d28/2y**	**ratio d28/3y**
**All ages**	1.88	0.70	0.36	0.30	**2.7**	**5.2**	**6.2**	164.20	28.82	11.09	10.55	**5.7**	**14.8**	**15.6**
**u4s**	11.71	7.60	0.04	0.00	**1.5**	**262.4**	**4383.3**	4.05	3.53	3.33	3.31	**1.1**	**1.2**	**1.2**
**sch**	2.78	0.69	0.20	0.18	**4.0**	**14.1**	**15.9**	77.13	21.78	7.48	7.38	**3.5**	**10.3**	**10.5**
**ado**	2.41	1.45	0.61	0.43	**1.7**	**4.0**	**5.5**	321.06	216.84	47.23	35.61	**1.5**	**6.8**	**9.0**
**adu**	1.75	1.05	0.89	0.88	**1.7**	**2.0**	**2.0**	186.35	143.92	50.78	32.89	**1.3**	**3.7**	**5.7**
**eld**	0.21	0.10	0.08	0.08	**2.1**	**2.6**	**2.6**	195.88	73.79	22.10	16.78	**2.7**	**8.9**	**11.7**

**IgG4**	**peak (d28)**	**[Ig]t=wk10**	**[Ig]t=2y**	**[Ig]t=3y**	**ratio d28/wk10**	**ratio d28/2y**	**ratio d28/3y**	**peak (d28)**	**[Ig]t=wk10**	**[Ig]t=2y**	**[Ig]t=3y**	**ratio d28/wk10**	**ratio d28/2y**	**ratio d28/3y**
**All ages**	0.27	0.13	0.09	0.08	**2.0**	**3.1**	**3.4**	0.25	0.11	0.08	0.08	**2.3**	**3.1**	**3.1**
**u4s**	0.97	0.72	0.05	0.01	**1.3**	**20.3**	**83.9**	0.13	0.10	0.01	0.00	**1.4**	**16.1**	**59.5**
**sch**	0.24	0.11	0.04	0.03	**2.2**	**5.8**	**7.5**	1.04	0.47	0.10	0.06	**2.2**	**10.3**	**18.1**
**ado**	0.48	0.16	0.08	0.07	**3.0**	**5.9**	**6.8**	0.17	0.11	0.09	0.09	**1.6**	**1.9**	**1.9**
**adu**	0.40	0.24	0.20	0.19	**1.6**	**2.0**	**2.0**	0.21	0.12	0.10	0.10	**1.7**	**2.1**	**2.1**
**eld**	0.05	0.04	0.04	0.04	**1.2**	**1.3**	**1.3**	0.12	0.08	0.06	0.06	**1.5**	**1.8**	**1.8**
